# Determinants of the interruption of exclusive breastfeeding at the 30th day after birth

**DOI:** 10.1590/1984-0462/2022/40/2021096IN

**Published:** 2022-06-10

**Authors:** Daniele Azevedo Kanan de Freitas, Thaymê Pires, Bruna dos Santos Willges, Letícia Daudt, Kimberli Dantas Käfer, Franciele da Silva Martins, Leandro Meirelles Nunes

**Affiliations:** a Hospital de Clínicas de Porto Alegre, Porto Alegre, RS, Brazil.; b Universidade Federal do Rio Grande do Sul, Porto Alegre, RS, Brazil.

**Keywords:** Breast feeding, Epidemiologic factors, Weaning, Cross-sectional studies, Multivariate analysis, Aleitamento materno, Determinantes epidemiológicos, Desmame precoce, Estudos transversais, Análise multivariada

## Abstract

**Objective:**

To estimate the prevalence of exclusive breastfeeding (EBF), introduction of water, herbal teas, or other milks, as well as to identify the factors associated with the interruption of EBF at the 30^th^ day after birth.

**Methods:**

This is a cross-sectional study using structured and pretested questionnaires applied to 310 mothers in two moments: in person, at the maternity ward; and at the time the infant was 30 days of age, by telephone call. Descriptive statistics and multivariate Poisson regression, following a multilevel hierarchical model according to the proximity to the outcome, were used to estimate the association between dependent and independent variables.

**Results:**

The maintenance of EBF at 30 days of age of the infant occurred in 85.2% of the sample, 1.6% receiving water, 11.5% herbal teas, and 8.2% other milk. Predictors for EBF interruption in the univariate analysis were the mothers’ return to work or study activities shortly after the baby's birth (IR 2.88; 95%CI 1.14–7.25) and the use of a pacifier (IR 3.29; 95%CI 1.52–6.22). The interruption of EBF was lower in the group of participants who received support from the infant's maternal grandmother (IR 2.71; 95%CI 1.11–6.56) and their partner (IR 4.78; 95%CI 1.90–12.06). After a multivariate analysis, only the use of a pacifier (IR 5.47; 95%CI 2.38–19.3) and the partner's support (IR 6.87; 95%CI 2.04–23.1) maintained the association with the outcome.

**Conclusions:**

The prevalence of EBF found in this study can be considered good, and future interventions aimed at increasing the duration of EBF in this population should take into account the participation of the partner and the reinforcement for not introducing the pacifier.

## RESUMO

**Objetivo:**

Estimar a prevalência de aleitamento materno exclusivo (AME), introdução de água, chás ou outros leites, bem como identificar os fatores associados à interrupção do AME aos 30 dias de vida.

**Métodos:**

Estudo transversal que utilizou questionários estruturados e pré-testados, aplicados a 310 mães em dois momentos: presencialmente, na maternidade, e aos 30 dias de vida da criança, mediante ligação telefônica. Estatística descritiva e regressão multivariada de Poisson, seguindo modelo hierárquico multiníveis conforme a proximidade com o desfecho, estimaram a associação entre as variáveis dependentes e independentes.

**Resultados:**

A manutenção do AME aos 30 dias de idade da criança ocorreu em 85,2% da amostra, e 1,6 % receberam água, 11,5 % chás e 8,2% outro leite. Preditores para a interrupção do AME na análise univariada foram o retorno das mães ao trabalho ou estudo logo após o nascimento do bebê (razão de incidência — RI 2,88; intervalo de confiança — IC95% 1,14–7,25) e o uso de chupeta (RI 3,29; IC95% 1,52–6,22). A interrupção do AME foi menor no grupo de participantes que recebeu apoio da avó materna do lactente (RI 2,71; IC95% 1,11–6,56) e do companheiro (RI 4,78; IC95% 1,90–12,06). Após a análise multivariada, apenas o uso de chupeta (RI 5,47; IC95% 2,38–19,3) e o apoio do companheiro (RI 6,87; IC95% 2,04–23,1) mantiveram associação com o desfecho.

**Conclusões:**

A prevalência de AME encontrada neste estudo pode ser considerada boa, e futuras intervenções que visem ao aumento da duração do AME nessa população devem levar em consideração a participação do companheiro e o reforço para a não introdução da chupeta.

## INTRODUCTION

According to the recommendation of the World Health Organization (WHO), breastfeeding (BF) should start in the first hour of life and is maintained exclusively for the first six months of the child's life, without the addition of other liquids and/or foods. After the sixth month of life, the introduction of healthy complementary food begins, and breastfeeding should be maintained for at least two years of life.^1^

These recommendations are based on scientific evidence of the benefits provided by BF.^2^ The following benefits stand out: protective effect against child morbidity and mortality and infections in general; less chance of developing allergic diseases; better cognitive, craniofacial, and oral motor development; and lower risk of developing chronic diseases in adulthood.^3–5^ For the mother, BF reduces the probability of occurrence of breast, ovarian, and uterine cancers, increases the interval between births, and provides faster uterine involution..^6^ In addition, it promotes the necessary bond for the mother-child binomial, creating the appropriate affective bonds for the child's psychological and emotional development.^3^ Many of these benefits are dose-dependent, that is, the longer the period during which the child receives milk or the mother breastfeeds, the greater the benefits of BF.^7^

However, in Brazil, exclusive breastfeeding (EBF) indicators are lower than the desirable outcome. The last national survey on the subject, published in 2008, when analyzing the set of Brazilian capitals and the Federal District, concluded that the prevalence of EBF in children under six months of age was 41%, while its average duration was only 1.8 month. It was also found that the unnecessary introduction of water occurred in 13.6% of children in the first month of life, while 15.3% of them received tea in the same period. The introduction of other milks accounted for 18% in the first month of life, and 48.8% of children received other types of milk between 120 and 180 days of life.^8^

More recently, in 2020, preliminary results from the National Survey of Food and Child Nutrition (*Estudo Nacional de Alimentação e Nutrição Infantil* – ENANI) showed an increase of more than 12 times in the prevalence of exclusive breastfeeding among children under four months of age compared with 1986, from 4.7 to 60%. Among children under six months of age, it increased by 42.8 percentage points, from 2.9 to 45.7% in these 34 years, which corresponds to an increase of 1.2% per year.^9^

The present study aims to estimate the prevalence of EBF, unnecessary introduction of water and/or teas and also of other milks, as well as to analyze the determining factors for the interruption of EBF at 30 days of life in children born in a hospital that is part of the Baby-Friendly Hospital Initiative. Thus, it intends to contribute to the planning of flows of collective actions that can interfere, directly or indirectly, in the promotion of EBF both at an institutional level and in terms of public health.

## METHOD

This is a cross-sectional study carried out at Hospital de Clínicas de Porto Alegre (HCPA), in the city of Porto Alegre, state of Rio Grande do Sul, Brazil. It is a general university hospital accredited as Baby-Friendly Hospital, with an average birth rate of approximately 3 thousand per year, with 99% of the attended mothers being users of the Brazilian Unified Health System. The target population consisted of postpartum mothers and their newborns, admitted to the rooming-in at the HCPA Obstetric Unit.

All mothers with full-term, healthy newborns (NB) at rooming-in, with baby's birth weight equal to or greater than 2500g, who did not give birth to twins, who started breastfeeding during hospitalization and were discharged with a medical prescription for EBF, until completing the calculated sample, were eligible to participate in the study. The pairs that, due to the mother's or the newborn's problems, had to be separated during hospitalization were excluded. Moreover, mothers who could not be contacted via telephone following the 30^th^ day of the baby's life were deemed as losses.

Data collection took place in two moments. Initially, after signing the informed consent form, between 24 and 48 hours of the NB's life, the mothers answered, through an interview, a questionnaire developed for this study, aiming to obtain sociodemographic information, data on prenatal care, childbirth, and previous experience of breastfeeding. The follow-up questionnaire was applied from 30 to 37 days of the infant's life, through an interview conducted by a researcher via telephone, and contained questions regarding breastfeeding, use of liquids other than breast milk, complementary feeding, sources of support for BF, and use of a pacifier.

A hierarchical regression model was developed, in which the variables are distributed into blocks according to their relationship with the outcome. The model suggested by Boccolini et al.^10^ was adopted, in which the hierarchy of the blocks considers the relationship of proximity of the exposure factors to the outcome. Therefore, the different variables were distributed into four blocks. The first includes maternal age (≥ or <20 years), maternal education (≥ or <8 years), family income (≥ or <1 minimum wage), residing with the infant's maternal grandmother and residing with the infant's paternal grandmother (yes or no); the second includes previous experience of breastfeeding (yes or no) and number of prenatal consultations (≥ or <6 consultations); the third one includes type of childbirth and sex of the newborn; and, in the fourth block, it is included the offer of infant formula at the maternity ward, use of pacifiers, return of the mother to study activities/work until the infant is 30 days of age, support from the partner, maternal grandmother, and paternal grandmother of the child during BF (all categorized as yes or no).

**Figure 1:**
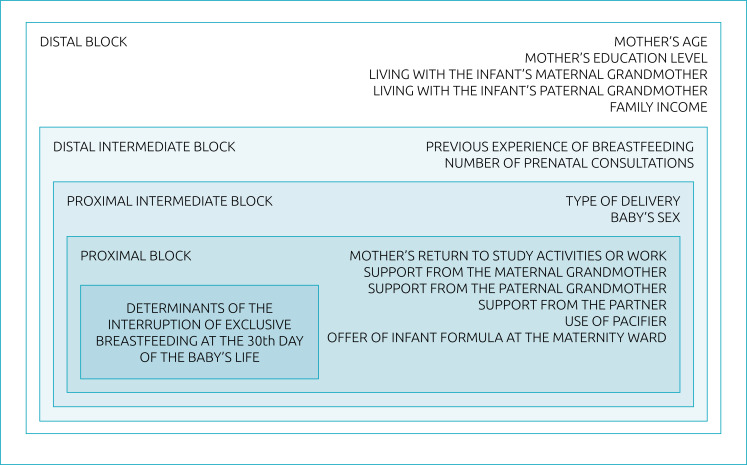
Conceptual hierarchical model used to identify factors associated with the interruption of exclusive breastfeeding at the 30^th^ day of the baby's life.

Adapted from Boccolini et al.^10^

First, analyses were performed to measure the association between the outcome and the variables of interest in each block, using univariate Poisson regression. The variables in the distal block that reached a significance level of p<0.20 in the univariate analysis were submitted to multivariate regression (intrablock analysis), with only variables that reached a significance level of p<0.10 remaining in the model for the adjustment of the distal intermediate block in the multivariate analysis of the distal block. Then, variables contained in the second block (distal intermediate level) that reached a significance level of p<0.20 in the univariate analysis were submitted to multivariate Poisson regression with the variables in the distal block that reached a significance level of p<0.10 in multivariate analysis; and so on. The model predicted that, once the variable reached a significance level of p<0.10 in the intrablock analysis, it would remain in the model until the end, adjusting the interactions between the variables in the other blocks as they were considered possible confounding factors (Figure 1). The degree of association between the several variables and the outcome was estimated using the incidence ratio (IR) and their respective 95% confidence intervals (95%CI), with an association with p<0.05 considered significant. Data processing was carried out using the *Statistical Package for the Social Sciences* (SPSS) software, version 21.0.

To calculate the sample size, the WINPEPI program, version 11.43, was used. Considering a test power of 80%, a significance level of 5%, an expected EBF rate of 61% in 30 days^8^, and an incidence density ratio of 1.3 as significant, according to the study by Venâncio et al.,^11^ the total sample size of 304 individuals was reached. Adding 10% for possible losses and refusals, the sample size should consist in 330 mother-NB binomials.

This study was conducted in accordance with the Health Research Standards established by Resolution No. 466/12 of the National Health Council, Brazilian Ministry of Health. The project was approved by the Research Ethics Committee of the HCPA (project No. 180419).

## RESULTS

Initially, 330 postpartum mothers-NB pairs were selected between March and June 2019, and the mothers were submitted to the initial questionnaire between 24 and 48 hours of the postpartum period. At the end of the application of the second questionnaire, 310 participants remained in the research. The remaining 20 patients were not included in the database and in the analyses due to failed attempt to contact them via telephone, after several attempts have been made.

The sociodemographic characteristics of the mothers, families and infants of the studied sample, according to the categorization of the blocks in the hierarchical model, are presented in Table 1.

**Table 1:** Descriptive characteristics of the variables studied and categorized according to the conceptual hierarchical model, according to proximity to the outcome (n=310).

		n (%)
Distal block		
	Maternal age <20 years	43 (13.9)
	Mother's level of education <8 years	77 (24.9)
	Residing with the partner	274 (88.4)
	Residing with the infant's maternal grandmother	71 (22.9)
	Residing with the infant's paternal grandmother	52 (16.7)
	Family income* <1 minimum wage	95 (30.6)
Distal intermediate block		
	I have previous experience of breastfeeding (>1 month)	170 (54.8)
	≥6 prenatal consultations	246 (79.3)
Proximal intermediate block		
	Vaginal childbirth	205 (66.1)
	Female newborn	160 (51.6)
Proximal block		
	I returned to studies or work in the 1^st^ month of the baby's life	28 (9.0)
	I had support from the infant's maternal grandmother	203 (65.5)
	I had support from infant's paternal grandmother	169 (54.5)
	I had support from my partner	256 (82.6)
	The baby used a pacifier	219 (70.6)
	I was offered infant formula at the maternity ward	33 (10.6)

Distal block: sociodemographic variables, maternal and family characteristics; Distal intermediate block: variables regarding the prenatal period; Proximal intermediate block: variables regarding childbirth, immediate postpartum period, and newborn characteristics; Proximal block: characteristics of nursing mothers and children;

* 1 minimum wage corresponded to US 190.13 when the study was carried out.

When initially questioned, 100% of mothers intended to exclusively breastfeed in the first six months of the baby's life. EBF maintenance at 30 days of life occurred in 85.2% of the sample, and 1.6% of the mothers started offering water; 11.5%, herbal tea; and 8.2% of the babies received other milks.

When analyzing questions on the use of pacifiers, 69.7% of the mothers reported having already had a pacifier at home at the time of the newborn's birth; 54.9% of mothers intended to offer a pacifier (data not shown in tables) and 70.6% of infants actually used it at 30 days of life.

Table 2 presents the univariate and multivariate analyses, testing associations between the interruption of EBF at 30 days of life and the selected variables. In the univariate analysis, there was a significant association between the outcome and the mothers’ return to work or study activities soon after the child's birth (IR 2.88; 95%CI 1.14–7.25) and the use of a pacifier (IR 3.29; 95%CI 1.52–6.22). The interruption of EBF was lower in the group of participants who received support from the infant's maternal grandmother (IR 2.71; 95%CI 1.11–6.56) and their partners (IR 4.78; 95%CI 1.90–12.06). It is observed that only variables from the proximal and distal blocks, and none of the two intermediate blocks, showed an association with the outcome. When performing the multivariate analysis, only partner's support (IR 6.87; 95%CI 2.04–23.1) and the use of a pacifier (IR 5.47; 95%CI 2.38–19.3) remained statistically significant.

**Table 2:** Univariate and multivariate analyses of factors associated with interruption of exclusive breastfeeding at the 30^th^ day of the baby's life. Analysis of variables submitted to the hierarchical Poisson regression model.

		n (%)	Univariate analysis		Multivariate analysis	
			p-value	IR (95%CI)	p-value	IR (95%CI)
Distal block						
	Maternal age <20 years	43 (13.9)	0.711	1.23 (0.40–3.81)	–	–
	Mother's level of education <8 years	77 (24.9)	0.345	1.53 (0.63–3.73)	–	–
	Residing with the infant's maternal grandmother	71 (22.9)	0.251	1.67 (0.69–4.06)	–	–
	Residing with the infant's paternal grandmother	52 (16.7)	0.474	0.60 (0.14–2.42)	–	–
	Family income <1 minimum wage	95 (30.6)	0.660	1.22 (0.49–3.02)	–	–
Distal intermediate block						
	Previous experience of breastfeeding	170 (54.8)	0.334	0.65 (0.27–1.55)	–	–
	Number of prenatal consultations <6	64 (20.7)	0.515	1.48 (0.75–3.69)	–	–
Proximal intermediate block						
	Type of childbirth — cesarean delivery	105 (33.5)	0.979	0.98 (0.40–2.44)	–	–
	Male newborn	150 (48.4)	0.170	0.53 (0.21–1.33)	–	–
Proximal block						
	Mother's return to study activities/work	28 (9.0)	**0.021**	**2.88 (1.14–7.25)**	0.463	1.82 (0.36–9.19)
	Support from the infant's maternal grandmother	203 (65.5)	**0.020**	**2.71 (1.11–6.56)**	0.532	0.67 (0.19–2.30)
	Support from infant's paternal grandmother	169 (54.5)	0.210	2.10 (0.65–6.71)	–	–
	Support from the partner	256 (82.6)	**0.002**	**4.78 (1.90–12.0)**	**<0.001**	**6.87 (2.04–23.10)**
	Use of pacifier	219 (70.6)	**0.005**	**3.29 (1.52–6.22)**	**<0.001**	**5.47 (2.38–19.30)**
	Offer of infant formula at the maternity ward	33 (10.6)	0.474	0.49 (0.71–3.40)	–	–

IR: incidence ratio; 95%CI: 95% confidence interval.

## DISCUSSION

With regard to the prevalence of EBF, the result of 85.2% achieved in the studied sample is higher than that reported by the II Research of Breastfeeding Predominance in Brazilian Capitals and Federal District, the last survey conducted nationwide, which found 60.6% of EBF at 30 days of baby's life in Porto Alegre, the city where the present study was carried out.^8^ Furthermore, the findings of this study concerning the offer of water, tea, and other milks — of 1.6, 11.5, and 8.2%, respectively — are below those found by the same study, which reported 7.3, 14.8 and 15.9% of mothers offering these liquids other than breast milk.^8^

It is worth emphasizing that this study was conducted at a Baby-Friendly Hospital, which is part of this initiative since 1997, in which the entire healthcare team is trained to promote EBF and its protection, having nurse consultants specialized in EBF at the rooming-in, and a telephone available 24 hours a day in such a way that mothers, after hospital discharge, can ask questions regarding breastfeeding. This could partly explain the good results achieved. In addition, the advances made towards the expansion of the practice of breastfeeding in Brazil in recent decades are undeniable. Although the identification of the factors that led to this improvement goes beyond the scope of this study, it is important, for the purposes of contextualization, to point out some changes in the evolution of the national policy on BF, as these factors may have influenced this behavior: massive BF campaigns with active engagement of public sectors and activists, national coordination of breastfeeding initiatives, national legislation on BF protection, expansion of maternity leave to four months in private sectors and six months in public sectors, encouragement to create breastfeeding rooms in the workplace, and regulation of advertisements for breastfeeding substitutes, among others.

Conversely, some women continued to offer water and/or herbal tea to their children, even though they received guidance at the hospital that this offer would be unnecessary. This reflects a deeply rooted cultural practice in the Brazilian population. The use of these liquids, particularly tea, represents a strong cultural issue worldwide and has been maintained based on popular beliefs such as to calm a restless child, for the purposes of hydration, or even to relieve an infant's colic.^12^

As for the determining factors for the interruption of EBF in the first month of life, most of previous studies assess the maintenance of BF for periods over 30 days, indicating as positive determinants for breastfeeding the support of the maternal grandmother and partner/father of the baby (provided that they are both favorable to breastfeeding) and pointing out negative associations with the use of pacifiers and of infant formula while still at the rooming-in, with the age of the parents (especially teenage mothers), and with the return to work, among other variables.^2,13–15^

The association with the infant's maternal grandmother support is widely researched and acknowledged.^15–17^ A systematic review has shown that maternal decisions can be influenced by the opinion of the child's maternal grandmother, which, when positive, can increase by 12% the probability of a mother to breastfeed; however, when negative, it can influence a reduction of up to 70% in the probability of breastfeeding.^15^ In the present study, the support of the infant's maternal grandmother was found to be significant in the univariate analysis; however, in the multivariate analysis, this association was not maintained. Perhaps this result is due to the fact that the infant's grandmothers give greater freedom to their daughters to play their roles as mothers. Regardless of this finding, the authors believe in the importance of including grandmothers, when present, in the guidelines on BF promotion and in their fundamental role as supporters.

Partner's support showed a significant association with the outcome in both analyses. A systematic review points out that the inclusion of partners in interventions aimed at the practice of breastfeeding improves the rates of initiation, duration, and exclusivity of breastfeeding.^18^ The following actions have a greater chance of success: actions that offer support during the prenatal period or immediately after birth; those carried out in person by trained professionals; those that are planned taking into account cultural characteristics; and those focused on information on how partners can support breastfeeding.^18,19^

The mother's return to work or study activities in the child's first month of life was significant in the univariate model, but not in the multivariate one, probably because mothers with these characteristics represent a small percentage of the studied sample, i.e., less than 10%. Nonetheless, this is a factor that must always be considered and that may be associated with the interruption of EBF, as the full-time presence of the mother is a basic condition for the maintenance of the practice, in addition to being necessary for the formation of the mother-child bond.

As for the use of pacifiers, the present study verified a high prevalence in relation to the last survey conducted in the Brazilian territory: 70.6 *versus* 42.6%.%.^8^ Moreover, the aforementioned survey showed a high prevalence in Rio Grande do Sul (53.7%), the state with the highest rate of pacifier use when compared with other regions of the country, reaching double the prevalence in the North region.^8^ Even though mothers are attended in a hospital part of the Baby-Friendly Hospital Initiative, according to which one of the steps is the guidance for not using pacifiers, it seems that mothers do not have this information emphatically or they prefer not to follow such step, which allows the authors to infer that the culture of using pacifiers is ingrained in the Brazilian population.

The use of pacifiers has been the subject of epidemiological surveys in order to identify possible benefits or harms when used in children, and it has been associated with a reduction in the duration of breastfeeding and an increased risk of the child developing acute otitis media, gastroenteritis, masticatory dysfunction, and dysocclusion.^20^ Conversely, a meta-analysis published in 2017 found a strong protective effect of pacifier use on sudden infant death.^21^ However, considering the concern that pacifier use may interfere with breastfeeding, confirmed by a systematic review that found a positive association between pacifier use and EBF interruption in observational studies,^22^ the introduction of this tool in breastfed babies with risk factors for sudden death should be postponed until breastfeeding is well-established, which usually occurs after the first month of life.^21,22^

The present study showed an association between pacifier use in the first month of life and EBF interruption in both univariate and multivariate analyses. The cross-sectional design of this study does not assist in elucidating the mechanisms involved in this interaction; nevertheless, four hypotheses to explain the association between reduced pacifier use and longer EBF duration are found in the literature: pacifier use alone can reduce breastfeeding duration; the introduction of pacifiers occurs due to difficulties in breastfeeding; the baby's personality and the mother-infant interaction make mothers use the pacifier as a way for the child not to latch on for so long, to calm down, or sleep more easily; and the profile of mothers and their families determines the option to breastfeed and avoid pacifiers.^20^

Other factors mentioned in the literature as determinants for the interruption of EBF in the first month of the child's life, such as male NB, low number of prenatal consultations, and mother's low level of education,^10^ were not statistically significant in this study, despite of male NB presenting p-value very close to statistical significance, indicating a possible tendency to early EBF interruption. When analyzing the different published studies, the authors infer that the present results may differ from those of other publications, as the determinants of EBF interruption may vary over time and according to the study location.

A possible limitation of the study could be the fact that the follow-up questionnaires were applied via telephone contact, in addition to being relatively extensive. The option for closed-ended questions with standardized answers and trained interviewers were the ways with which the authors sought to alleviate these limitations.

It can be concluded that the recognition of factors associated with the interruption of EBF is of great importance for the development of policies aimed at increasing BF rates. According to the results, EBF rates in the first month of the baby's life in a sample of infants born in a hospital part of the Baby-Friendly Hospital Initiative were good, and the determining factor related to the interruption of this practice was the use of a pacifier. Furthermore, EBF interruption was lower in the group of participants who received support from their partner.

The findings of this study may contribute to the implementation of strategies for promoting EBF, as well as to notify mothers, their families, and healthcare professionals of the possible determinants identified in this research.
